# The Role of Birch Tar in Changing the Physicochemical and Biocidal Properties of Polylactide-Based Films

**DOI:** 10.3390/ijms23010268

**Published:** 2021-12-27

**Authors:** Agnieszka Richert, Ewa Olewnik-Kruszkowska, Grażyna B. Dąbrowska, Henryk P. Dąbrowski

**Affiliations:** 1 Department of Genetics, Faculty of Biology and Veterinary Science, Nicolaus Copernicus University in Toruń, 87-100 Torun, Poland; browsk@umk.pl; 2 Department of Physical Chemistry and Physicochemistry of Polymers, Faculty of Chemistry, Nicolaus Copernicus University in Toruń, 87-100 Torun, Poland; olewnik@umk.pl; 3 Laboratory of Dendrochronology, Archaeological Museum in Biskupin, 88-410 Gasawa, Poland; hpdabrowski@biskupin.pl

**Keywords:** polylactide, agriculturalfilms, antibacterial properties, birch tar

## Abstract

The objective of this study was to produce bactericidal polymer films containing birch tar (BT). The produced polymer films contain PLA, plasticiser PEG (5% wt.) and birch tar (1, 5 and 10% wt.). Compared to plasticised PLA, films with BT were characterised by reduced elongation at break and reduced water vapour permeability, which was the lowest in the case of film with 10% wt. BT content. Changes in the morphology of the produced materials were observed by performing scanning electron microscopy (SEM) and atomic force microscopy (AFM) analysis; the addition of BT caused the surface of the film to be non-uniform and to contain recesses. FTIR analysis of plasticised PLA/BT films showed that the addition of birch tar did not change the crystallinity of the obtained materials. According to ISO 22196: 2011, the PLA film with 10% wt. BT content showed the highest antibacterial effect against the plant pathogens *A. tumefaciens*, *X. campestris*, *P. brassicacearum*, *P. corrugata*, *P. syringae*. It was found that the introduction of birch tar to plasticised PLA leads to a material with biocidal effect and favourable physicochemical and structural properties, which classifies this material for agricultural and horticultural applications.

## 1. Introduction

The presence of pathogenic microorganisms in the environment continues to be a serious problem. Ways are being sought to protect human, animal and plant organisms against the negative effects of pathogens. The multiplication of bacteria contributes to the spread of diseases and the destruction of agricultural crops [1]. This group of microorganisms mainly includes strains such as *Bacillus* sp., *Listeria monocytogens*, *Salmonella* sp. [2,3]. Due to the possibility of undesirable contact between microorganisms and agricultural products, effective and safe methods of combating undesirable bacteria are being developed [4]. For this reason, the agricultural industry is constantly looking for new, innovative solutions (including bactericidal films) to control the fight against pathogenic microorganisms. Furthermore, these films should be environmentally friendly, should not show phytotoxicity, and should be biodegraded after the end of their service life. Polylactide (PLA), due to its biodegradability, easy availability, and relatively good mechanical strength, is one of the polymers most commonly used for such purposes. The demand for new, eco-friendly composites is increasing every year. The production of films with antibacterial properties involves the direct introduction of biologically active substances into the polymer matrix [5,6]. Many scientists have reported results of detailed research on bactericidal films based on polylactide (PLA) with the addition of the following: cinnamon and garlic oil [7,8]; clove oil [6,9]; lemongrass, rosemary, or bergamot oil [10]; tea tree oil [11]; bacteriocins [12]; and polyhexamethylene guanidine [13,14,15]. Plasticisers are often used to increase the flexibility of polymer films [7]. Poly(ethylene glycol) (PEG) is one of the most commonly used substances for polylactide plasticization [4,16]. The liquid fraction called tar is a characteristic product of the distillation of wood and birch bark. Dry distillation of birch bark yields a particularly large amount of birch tar (BT), which can amount to about 15% BT is a viscous liquid with a density of 0.925–0.950 g cm^−3^ and a characteristic odour. The chemical compounds included in the BT are mainly phenol derivatives (guaiacol, creosote, pyrocatechin), betulin, benzene, xylene, phytoncides, organic acids, and resin substances. Today, BT is used primarily in the medicine, veterinary medicine, pharmaceutical, and cosmetic industries as an ingredient of, for example, soaps, ointments and oils. Tar has many properties, including bactericidal, antiseptic, and anti-inflammatory and is used in medicine [17,18]. Due to its strong antiseptic effect, this compound is used in veterinary medicine for the treatment and care of hooves (concentration range 1–30%) [18]. BT contains substances with a stimulating effect on the reconstruction of damaged hoof material (frog thrush is a common disease of horse hooves) [19]. In accordance with international regulations (EC 1996, number 1492), the environmental impact of chemicals must be assessed prior to their intended use in the field. Research has shown [19] that aquatic organisms are somewhat sensitive to BT oil, but at the same time suggests that it poses no serious threat to aquatic biota. If BT oil is not used in the immediate vicinity of bodies of water, no special precautions are required [19].

The objective of this study was to produce and characterise polylactide-based materials with antibacterial properties. We assumed that adding BT to plasticised PLA with 5% wt. PEG, which is known for its antibacterial properties, would also impart these properties to the new materials. Plasticised PLA materials with 1, 5, and 10% wt. BT content were analysed in terms of surface structure, barrier properties, elongation at break, and antibacterial properties.

## 2. Results and Discussion

### 2.1. FTIR-ATR

FTIR-ATR spectroscopy was applied to determine the effect of 1, 5, and 10% wt. BT on the structure of the obtained plasticised PLA materials. The structure of PLA and of PLA with an addition of poly(ethylene glycol) is well known and extensively described in the literature [4,20]. For this reason, only the most significant bands have been listed in Table 1. 

**Figure 1:**
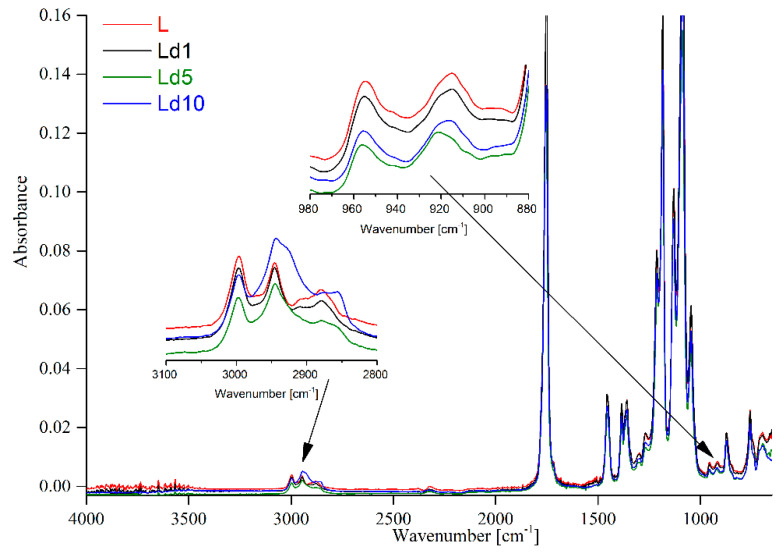
FTIR-ATR spectra of plasticised PLA with and without of BT.

However, taking into account the obtained spectra presented in Figure 1, it is clear that after the introduction of BT into plasticised PLA matrix, the only changes observed are indicated by the bands at 2850 cm^−1^ and 2927 cm^−1^.

The composition of BT has previously been described by Fagerna et al. [21], who indicated that BT may be a promising source for biological pesticides. Chemical composition was also discussed in the work of Vladimirov [22], where 27 different compounds were identified, accounting for 93.5% of the total composition.BT is a substance that does not dissolve in water, so BT films absorb water to a lesser extent and also have reduced water vapour permeability. Based on the works cited, it can be assumed that the composition of BT is very complex and can cause problems during FTIR-ATR analysis. The listed bands can be assigned to the -CH and CH_2_ groups. Moreover, higher BT content results in slight changes in the intensity of bands at 1130 cm^−1^, 1183 cm^−1^ and 1212 cm^−1^, which can be assigned to the C-O-C and -C-O groups, respectively. Another fact to be taken into consideration is that new bands were not observed (Figure 1). For this reason, it can be assumed that no new bands are formed between the polymer matrix and the studied additive introduced into the structure. It is well known that bands at 915 cm^−^^1^ and 955 cm^−^^1^ belong to the amorphous and crystalline phase, respectively [4]. As can be seen, the relationship between the intensity of both bands is constant (Figure 1). For this reason, the results suggest that the addition of birch tar does not influence the crystallinity of the obtained materials.

### 2.2. Assessment of Film Morphology

**Figure 2:**
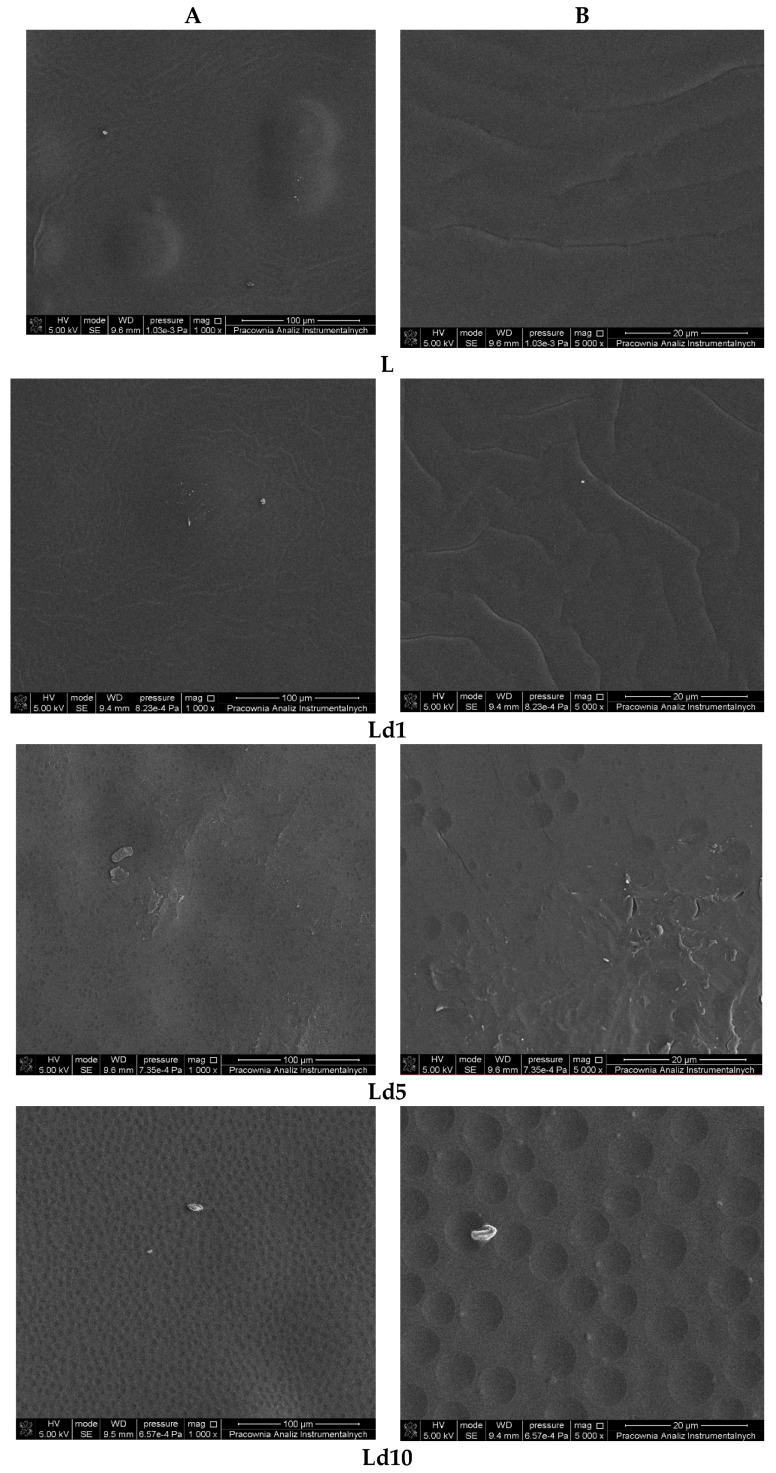
SEM analysis of the surface of plasticised PLA (L) and plasticised PLA with BT materials (Ld1–Ld10) materials, magnifications at 1000× (**A**) and 5000× (**B**).

This study focused on plasticised PLA materials with an addition of BT as an antibacterial agent. Scanning electron microscopy (SEM) and atomic force microscopy (AFM) were used to analyse the surface morphology of the obtained polymeric films. Figure 2 shows SEM images of the L, Ld1–Ld10 materials.

As can be observed, the material consisting of polylactide with the addition of plasticiser in the form of poly(ethylene glycol) is characterised by a rippled surface with irregularities indicating the limited miscibility of the used polymers used.

Surface morphology and topography are among the critical factors that can significantly influence adhesion between polymeric film and the cells of microorganisms [23,24]. It is well known that bacterial adhesion often determines potential applications of materials and indicates if a particular polymeric film can be used in medical applications and agriculture. Based on extensive research, it has been established that there is no single topographic pattern of materials characterised as favourable in terms of bacterial adhesion [23]. Moreover, it has been stated that, alongside surface topography, the shape and size of bacteria significantly influence the interaction between the surface of a polymeric material and bacteria [23].

### 2.3. Examination of Surface Topography of Plasticised PLA Films with and without BT

**Figure 3:**
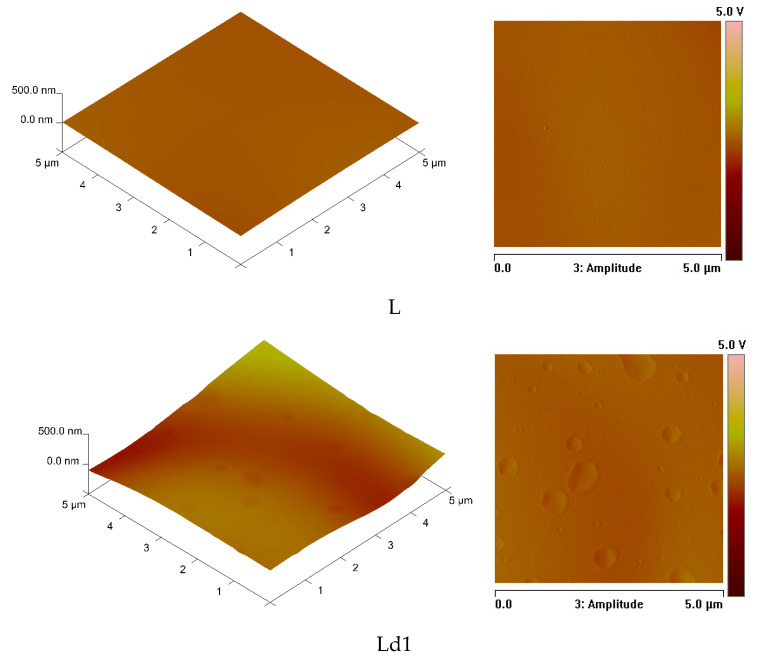

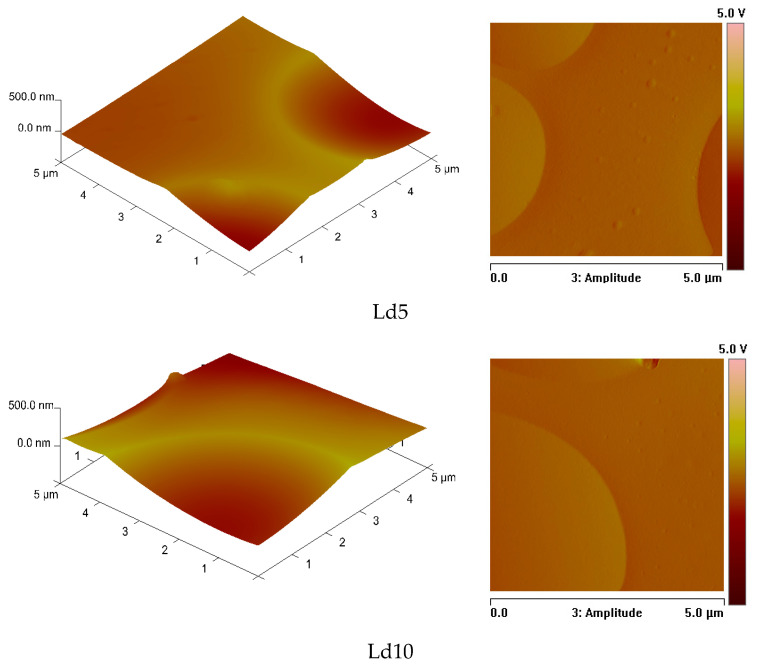
Surface images of the obtained plasticised PLA (L) and plasticised PLA with BT (Ld1–Ld10): photos taken using AFM.

The introduction of BT into the polymeric matrix is not sufficient to ensure the antibacterial effectiveness of the obtained materials. The second factor that can significantly affect the antibacterial properties of plasticised PLA films is the roughness of the surface. To assess the roughness parameters of the materials, AFM microscopy seems to be the most adequate technique. Figure 3 shows three-dimensional images of the surfaces of the obtained materials and two-dimensional images of the amplitude. The formation of circular recesses on the analysed surfaces is clearly visible.

Upon analysis of Figure 3, obtained by AFM, it can be concluded that, as the amount of BT increases (1, 5, and 10% wt.), plasticised PLA polymer shows correspondingly greater losses on the film surface.

The AFM analysis also allowed us to establish the R_a_ values (mean arithmetic deviation of the profile from the mean line) and R_q_ (mean square deviation of surface roughness). The values of the aforementioned roughness parameters are presented in Table 2.

**Figure 4:**
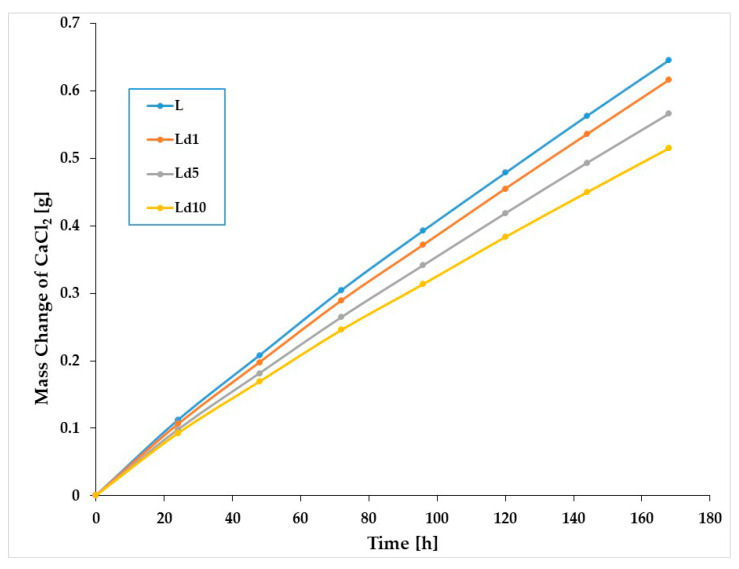
Changes in CaCl_2_ mass versus time during analyses of WVPR of the studied materials.

**Figure 5:**
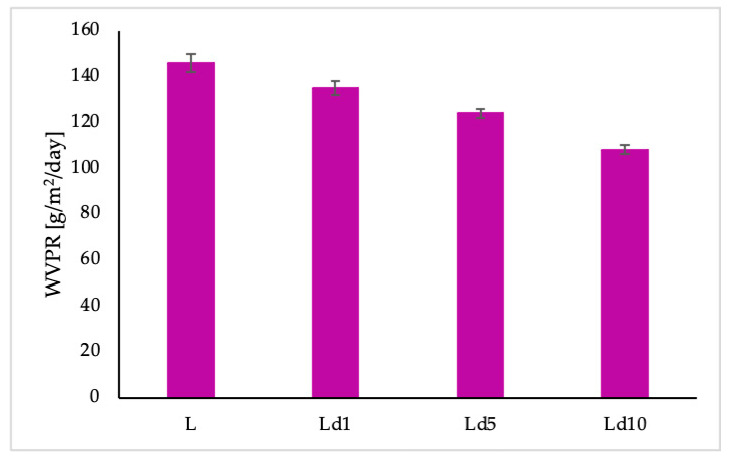
Water vapour transmission rate of the studied polymeric films.

In our research, the formation of PLA and PEG and/or BT phases was observed (Figure 4 and Figure 5). The introduction of BT into PLA-PEG system significantly affects the morphology of the obtained materials. The surface of a plasticised PLA film with an addition of 1% wt. of BT is covered with more cracks than the surface of a film with a polymer matrix. The introduction of a higher content (5 and 10% wt.) of BT into plasticised PLA results in the formation of holes, cavities and pores evenly distributed across the surface. Furthermore, it should be emphasised that the number and size of cavities observed on the analysed surfaces increased with the increase in the amount of BT added to PLA. Interestingly, in addition to the increase in the number of cavities, their size also significantly increased. The content of many different compounds present in the tar makes it difficult to form a homogeneous mixture with polylactide. These compounds include sugars, betulin, terpenoids, organic acids and resinous substances. The recorded SEM and AFM images allowed us to establish the diameters of the hollows that formed on the studied surfaces. As mentioned above, the size of the recesses significantly depends on the concentration of the biocidal agent. The largest diameter of the cavities was observed for sample Ld10 and measured 7.5 µm. Based on the AFM results, the depth of the cavities was also analysed. In the case of Ld1, Ld5 and Ld10 films, the cavities were concave to an extent of: 52 nm, 74 nm and 102 nm, respectively. The results lead to the conclusion that the diameter and depth of the hollows increase significantly with increasing amount of the antibacterial agent.

The values of the R_a_ and R_q_ parameters confirm that the size of cavities on the surface of analysed films of plasticised PLA with BT increases with increasing BT content in the materials (Table 2). Moreover, it should be emphasised that the R_q_ values justify the conclusion that the formed circles are evenly spread on the surface regardless of the amount of antibacterial agent introduced.

### 2.4. Evaluation of Mechanical Properties of Films

It is commonly recognised that the mechanical properties of films are extremely important [15,25]. To establish the impact that different amounts of the biocidal agent (in the form of BT) had on mechanical properties, Young’s modulus (E) and elongation at break (ε) were determined. The results of the mechanical properties are shown in Table 3.

The addition of BT to the PLA/PEG solution allowed to be obtained materials with higher Young’s modulus and lower elongation at break than the control sample consisting of PLA and PEG (L) (Table 3). As the BT content in the polymer matrix increased, the elongation at break value gradually decreased. This decrease was, respectively, as follows: 1.9, 2.1, and 2.7% for the Ld1, Ld5 and Ld10 films, as compared to the control sample L. Therefore, we note that BT films were characterized by almost two times lower elongation at break values, while, for the Ld10 film, this parameter was almost three times lower. The authors of research studies on biodegradable polymeric materials containing various biocides (but not BT) have reached similar conclusions [23,26,27].

Based on the obtained results it was established that the decrease in the elongation at break value, after the addition of BT, can indicate that birch tar has an antagonistic effect on the polymer chain movement capacity. It is consistent with results described in the work of Grabska-Zielińska et al. [28], where the materials consisting of PLA, PEG, and olive leaf extract were studied. The research of Pluta and Piórkowska [29] showed that the physical properties of PLA, which has a low plastic deformation capacity, are changed by mixing this polymer with a PEG-b-PPG-b-PEG copolymer with different PEG content (10 and 40%). The authors obtained a significant increase in the ductility of the PLA mixture with 10% wt. copolymer [29] and emphasize that the copolymer dispersion in the PLA matrix plays an important role in the deformation mechanism of the mixtures [30,31,32]. In turn, Baiardo et al. [33] showed that for effective plasticisation it is important to lower the glass transition temperature of a mixture of PLA with a plasticizer to at least 35 °C. Moreover, it should be emphasized that in the case of the Ld1, Ld5 and Ld10 samples, the Young’s modulus values are, respectively, 2.16, 17.1 and 19.8% higher than in the control, plasticised L film. The highest Young’s modulus was observed for the film Ld10 and amounted to approximately 1314 MPa. The authors observed the same trend for the value of Young’s modulus when querticin was added to PLA-PEG system, but they did not notice a decrease in the value of elongation at break [4].

### 2.5. Water Vapour Permeation Rate (WVPR)

The WVPR for the tested films was checked. Figure 4 shows the changes in CaCl_2_ mass over 7 days of permeation analysis.

The studied materials (plasticised PLA with and without BT) show a clear reduction in water vapour permeability after the addition of BT. Permeability of water vapour decreased with increasing BT content in the polymer matrix.

The barrier properties of materials meant for use in different fields are very important and significantly influence their application. It has been established that biodegradable polymers such as polylactide have relatively high water vapour permeability due to their polarity [34]. The addition of a nanofiller into polylactide can enhance the resistance to water vapour permeation [27,35,36]. It has also been found that a layer-by-layer coating of polylactide with chitosan and cellulose nanocrystals and PLA with PHB yields biomaterials with significantly improved barrier properties [37,38]. Water vapour permeability has also been evaluated by Qui et al. [10], where different essential oils were introduced into the polylactide matrix as antibacterial compounds. It was proven that the type of essential oils significantly affects the WVPR value. Moreover, it was indicated that the differences are caused by the different composition of the antibacterial additives used in the study. In the current work, the antibacterial compound was in the form of viscous liquid undissolved in water. Tar consists of compounds such as phenol, guaiacol, toluene, cresol, pyrocatechol, phenanthrene, sesquiterpenes, chrysene, betulin, benzene, xylene, volatile organic acids, and resin substances. The highest reduction in WVPR with respect to PLA was observed for the Ld10 sample and amounted to 26%. Based on the results, the improved resistance to water vapour transmission is a potentially valuable feature for materials used in horticultural and agricultural products.

### 2.6. Antibacterial Activity of Plasticised PLA Films with BT

The results of bactericidal properties of plasticised polylactide films with BT are evaluated in Table 4.

Regardless of the bacterial strain used, the best antibacterial effect was recorded for the Ld10 film. Thus, the higher the BT content used to produce the film, the greater the antibacterial effect obtained. BT shows strong antibacterial properties due to the compounds present in it, such as the following: phenol, betulin, cresol, chrysene, sterols, triterpenoid esters, triterpenoids, acyl lipids [17,18,39]. The degree of sensitivity of the strains to the bactericidal film is as follows: *A. tumefaciens* ≥ *X. campestris* ≥ *P. brassicacearum* ≥ *P. corrugate* ≥ *P. syringae*.

All analysed films show very good antibacterial effectiveness against the bacterial strains of *A. tumefaciens* and *X. campestris*. The reduction in the number of viable bacterial cells (R) on the surface of this film exceeds the value of 2. In this study, a hitherto forgotten BT with a wide range of applications was tested. In the past, tar was a compound used in everyday life. Today, it is used primarily in the pharmaceutical and cosmetic industries as an ingredient of, i.a., soaps, ointments and oils [21]. Tar has many properties, including bactericidal, antiseptic and anti-inflammatory [17,22,40]. In our research, its antibacterial effects were tested.

Studies by Royer et al. [41] indicate that using methanol and ethanol as solvents, it is possible to extract polar compounds from birch tar; these include polyphenols, sugars and some organic acids, which account for a significant part of the total extract content. In turn, Krasutsky [42] showed that less polar solvents (such as chloroform and hexane), extract waxes and non-polar compounds (such as triterpenoids) are present in 20–35% of white birch bark. During the production of plasticized PLA film with BT, chloroform is used as a solvent, which at the same time enables the extraction of substances from BT, including those with antibacterial properties. In the conducted research, tar obtained from birch bark was used to increase the chance of producing new materials with antibacterial properties. It is well known, that the bark is the outer part of the tree, and thus the first barrier protecting the tree from unfavorable biotic and abiotic factors. Therefore, tree bark contains more metabolites with antimicrobial properties than does wood. A study by Omar et al. [43] has shown that broadleaf bark extracts had a stronger inhibitory effect against gram-positive and gram-negative bacterial strains than did wood extracts. In most studies, the antimicrobial activity of white birch extracts extracted using hexane (a less polar solvent) is associated with the high contents of non-polar triterpenoids, e.g., betulin and lupeolu [42,43,44]. Non-polar phenolic compounds and flavonoids are believed to be responsible for antimicrobial activity [41,45]. Bordeoux et al. [46] has shown a strong antimicrobial effect of acid-base extracts, specific for alkaloids obtained using chloroform or hexane. According to Kedzia et al. [47] the complex mechanism of antimicrobial action can be attributed to the synergism between flavonoids, hydroxy acids and sesquiterpenes [47].

Research on wood tar has also been undertaken by Shimizu et al. [48]. The scientists tested 41 compounds against the pathogenic strain of *Legionella pneumophila* that causes respiratory diseases. One of the most effective natural killing compounds for *L. pneumophila* was found to be birch tar oil (BTO). The MIC value for BT was 27.2 µg mL^−1^ (0.0024%). BTO was tested not only for its bactericidal activity but also as a slug repellent. The results of Lindqvist & Lindqvist [40] proved that BTO, especially when mixed with Vaseline^®^, acted as an excellent long-term mollusc repellent. Hagner et al. [11] also tested BTO, but for its effect on soil organisms and plants [11]. They have proved that the adverse effects of BTO on the soil environment are insignificant and short-lived. Other chemical products used as herbicides are much more harmful to soil organisms and plants, as compared to BTO. It has been demonstrated that BTO can be used as a natural herbicide. Hagner et al. [11] also undertook studies showing the effects of BTO on aquatic organisms. The bacterium *Vibrio fischeri* was found to be the most sensitive, and the snail *Lymnea* sp. was the least sensitive. The results of Hagner et al. [11] confirmed that BTO does not significantly harm aquatic organisms. To summarise, BT has proved itself to be an effective bactericide against pathogenic strains. Our research has confirmed the antibacterial properties of BT even after incorporation into the polymer matrix.

## 3. Materials and Methods

### 3.1. Materials

Polylactide (PLA) type 2003D type (Ingeo™ Biopolymer 2003D, Nature Works LLC, Blair, NE, USA) with a melt flow rate of 5–7 g 10 min^−^^1^ (2.16 kg; 190 °C) and a density of 1.24 g cm^−3^ in the form of pellets was used to prepare polymer solutions [49]. Birch tar (BT) (d), a dark, oily solid with a characteristic pungent odour, is a natural product obtained by dry distillation of birch bark (*Betula pendula* Roth). The chemical composition is very complex and not fully understood (Poland) [49]. Poly(ethylene glycol) (PEG) with Mw = 1500 g mol^−1^ (Sigma-Aldrich Ltd., Poznań, Poland) was used as a plasticiser. Chloroform (Chempur, PiekaryŚląskie, Poland) was used as a solvent [49]. The obtained materials (plasticised PLA with BT) were characterised in terms of biodegradation properties in the work of Richert et al. [49].

### 3.2. Preparation of Films

The examined films were prepared using a laboratory method. Polylactide pellets were dissolved in chloroform in an attempt to obtain a 3% (*w*/*v*) polymer solution [50]. Subsequently 1, 5 or 10% wt. of BT (in the form of an oily solid) was added to the PLA/PEG solution. In total, 5% wt. PEG was introduced into the solutions to prepare plasticised PLA films. To obtain PLA-based materials, 50 mL of the prepared mixture was poured onto glass Petri dishes (14.5 cm in diameter) and left for 3 days to form a polymer film [10,11,28,29,30,31,32,33,34,35,36,37,38,39,40,41,42,43,44,45,46,47,48,49,50]. The thickness of films was measured using an electronic thickness gauge (type 0.001/0–12.7 mm, Poland). It ranged from 0.075 to 0.080 mm.

The film marked as L consists of polylactide and PEG. The plasticised PLA material containing 1, 5, and 10% wt. of BT was denoted as Ld1, Ld5, Ld10 (Table 5).

### 3.3. Mechanical Properties

The mechanical properties of the plasticised PLA films with and without BT were analysed using an Instron 1193 machine (Instron Corp., Canton, OH, USA) test according to the PN-EN ISO 527-1, -3 standard [51,52]. Ten specimens were used for the tests, and the arithmetic mean of these measurements was taken as the final test result. The film shapes were placed in holders such that the longitudinal axis of the film coincided with the axis of the measuring holders of the testing machine. The crosshead speed was 20 mm min^−1^ with an applied force of 100 N. The results were used to determine Young’s modulus (E) and elongation at break (*ε*).

### 3.4. Water Vapour Permeation Rate

Based on the method described in the literature, the water vapour permeation rate (WVPP) of polylactide with and without BT materials was determined [11]. In the first stage, calcium chloride was dried at 110 °C for 48 h. In the second stage, an appropriate amount of CaCl_2_ was introduced into a container with the analysed polymeric films and tightly sealed. All containers were placed in a desiccator under controlled environmental conditions at 30 °C and 75% relative humidity. Changes in CaCl_2_ mass were then recorded every 24 h for 7 days. Measurements were performed in triplicate for each analysed sample. The slope of the obtained lines was determined to calculate the WVPR. The water vapour permeation rate was evaluated using the following Equation (1) [4,15,37]:

(1) $$WVPR=\frac{Slope\;of\;the\;straight\;line}{Surface\;area\;of\;the\;film}\left[\frac{g}{m^{2}\cdot day}\right]$$

### 3.5. Fourier Transform Infrared Analysis

The Fourier transform infrared analysis of all studied materials was performed using spectrometer Nicolet iS10 (Thermo Fisher Scientific, Waltham, MA, USA). Spectra were recorded in the frequency range of 500–4000 cm^−^^1^ at a resolution of 4 cm^−^^1^ and scanned 64 times. All spectra were analysed using the OMNIC 7.0 software (Thermo Fisher Scientific, Waltham, MA, USA).

### 3.6. Scanning Electron Microscopy

The morphology of the plasticised PLA films with and without BT was studied using a Quanta 3D FEG scanning electron microscope (SEM, FEI Company, Hillsboro, OR, USA). Photographs of the topography of the samples were taken using an SEM detector at 1000× and 5000× magnification. Prior to each analysis, the surfaces of the studied materials were sprayed with a layer of gold.

### 3.7. Atomic Force Microscopy

An atomic force microscope (AFM, NanoScopeMultiMode, Veeco Metrology, Inc., Santa Barbara, CA, USA) was employed for roughness analysis of the plasticised PLA materials (L, Ld1, Ld5, Ld10). All analyses were conducted in air at room temperature. Roughness parameters were calculated using the Nanoscope software for sample areas measuring 5 µm × 5 µm.

### 3.8. Antibacterial Properties of Films

To check the antibacterial properties of the film (L, Ld1–Ld10), analyses were performed in accordance with the ISO 22196:2011 standard [53]. Five agricultural gram-negative bacteria strains were used in the study: *A. tumefaciens*, *X. campestris*, *P. corrugata*, *P. brassicacearum*, *P. syringae* (strains from the collection of the Department of Genetics, Nicolaus Copernicus University in Toruń). The analysis was carried out in three repetitions for each of the analysed samples. Table 6 describes the antibacterial efficacy criteria.

Specified amounts of bacterial cells (10^6^) were applied onto control films of plasticised PLA (L) and test films (Ld1, Ld5, Ld10). After 0 h (for the reference sample) and 24 h for the reference sample and test samples, bacteria were retrieved from the surface of the films and placed in a neutralising solution. The number of cultured cells was then determined by placing them in a PCA (Plate Count Agar, Oxoid Company, Nepean, ON, Canada) medium used to determine the total bacterial growth of a sample. Incubation of microorganisms on plates containing the medium was carried out for 48 h at 35 °C. Antibacterial activity (R) was determined using the following equation:

*R* = (*Ut* − *Uo*) − (*At* − *Uo*) = *Ut* − *At* (2)

where:

*R* (log_10_ reduction)—the difference between the logarithm of the average number of CFUs (colony-forming units) on reference samples after 24 h, and the logarithm of the average *CFU* on the test samples; *R* is antibacterial activity.

*Uo*—log_10_ average of the number of living bacteria (cells/cm^2^) recovered from the reference sample immediately after inoculation (0 h).

*Ut*—log_10_ average of the number of living bacteria (cells/cm^2^) recovered from the reference sample after 24 h from inoculation.

*At*—log_10_ average of the number of living bacteria (cells/cm^2^) recovered from the test sample after 24 h from inoculation [53].

## 4. Conclusions

The research has shown that the inclusion of BT in polylactide matrix plasticised with PEG makes it possible to obtain films with antibacterial properties. The advantage of the obtained films is their antibacterial activity against plant pathogens, especially *A. tumefaciens* and *X. campestris*. The surface of the film structure was observed to be heterogeneous in the films produced with BT, and this probably favours the adhesion of bacterial strains to the film and thus increases the influence of the compounds contained in the tar on bacterial cells, thereby inhibiting their growth. On the other hand, the enhanced barrier properties found for plasticised PLA films with BT predispose them as materials for potential use in agriculture or horticulture to protect crops against plant pathogens. The proposed solution provides an opportunity to reduce the use of plant protection products in crops.

## 5. Patents

Richert, A., Dąbrowska, G.B., Dąbrowski, H.P., 2020. Bactericidal polylactide film and the method of its preparation. Patent Application P.433979 (in Polish).

## Figures and Tables

**Table 1:** Positions of characteristic bands present in the studied polymeric films.

Group	Positions of Characteristic Bands (cm^−1^)
-OH	3600–3000
-CH stretching ((-CH_3_(asym) and -CH_3_(sym))	2996 and 2947
-CH and -CH_2_	2880
-C=O	1763
-CH3	1457
-CH deformation and asymmetric bands	1386 and 1360
C-O stretching modes of the ester group	1212 and 1183
-C-O-C	1085
C-COO stretching vibration	873
deformation vibration of CO	756

**Table 2:** Roughness parameters of plasticised PLA and plasticised PLA with BT (Ld1–Ld10).

Sample	R_q_ [nm]	R_a_ [nm]
L	4.7	3.5
Ld1	43.6	32.9
Ld5	55.5	43.9
Ld10	73.9	62.6

**Table 3:** Mechanical properties of plasticised PLA-based films with and without of BT with ± standard deviation (SD).

Sample	Young’s Modulus (E) ± SD MPa	Elongation at Break (ε) ± SD %
L	1097.4 ± 67.3	15.8 ± 0.8
Ld1	1121.1 ± 51.1	8.2 ± 0.5
Ld5	1284.7 ± 26.2	7.4 ± 0.3
Ld10	1314.6 ± 91.4	5.7 ± 0.3

**Table 4:** Antibacterial activity of plasticised PLA and plasticised PLA with the addition BT against plant pathogens. Note: (-) not determined.

Quantitative Assessment of Activity Concentration of Starting Inoculum 2.54 × 10^5^					
Sample Description	Number of Bacteria Recovered	Log Value	R	% Reduction	Antibacterial Efficacy
*A. tumefaciens*					
L	4.60 × 10^5^	5.7	---	---	----
Ld1	2.98 × 10^1^	1.5	4.2	>99.9	very good
Ld5	2.98 × 10^1^	1.5	5.6	>99.9	very good
Ld10	<2.00 × 10^1^	<1.3	>5.8	>99.9	very good
*X. campestris*					
L	4.60 × 10^5^	5.7	---	---	----
Ld1	1.30 × 10^3^	3.1	4.0	>99.9	very good
Ld5	1.10 × 10^1^	3.0	4.1	>99.9	very good
Ld10	2.56 × 10^1^	1.4	4.3	>99.9	very good
*P. brassicacearum*					
L	1.34 × 10^7^	7.1	---	---	---
Ld1	3.86 × 10^4^	3.8	1.8	92.0	satisfactory
Ld5	2.08 × 10^3^	3.3	3.8	>99.9	very good
Ld10	1.32 × 10^3^	3.1	4.0	>99.9	very good
*P. corrugata*					
L	1.34 × 10^7^	7.1	---	---	---
Ld1	4.56 × 10^4^	4.4	1.7	91.0	satisfactory
Ld5	2.00 × 10^3^	3.1	3.5	>99.9	very good
Ld10	1.21 × 10^3^	2.7	3.7	>99.9	very good
*P. syringae*					
L	1.34 × 10^7^	7.1	---	---	---
Ld1	4.16 × 10^4^	4.6	1.1	91.0	satisfactory
Ld5	5.52 × 10^4^	4.8	2.2	91.0	very good
Ld10	4.08 × 10^3^	4.3	2.8	>99.9	very good

**Table 5:** Symbols and composition of individual samples.

Symbol Sample	Sample Composition		
	PLA [g]	Birch Tar [g]	PEG [g]
L	100.0	-	5.0
Ld1	100.0	1.0	5.0
Ld5	100.0	5.0	5.0
Ld10	100.0	10.0	5.0

**Table 6:** Antibacterial efficacy criteria (ISO 22196, 2011).

Antibacterial Activity R, log	Decrease in Number of Microorganisms	Antibacterial Efficacy
<1.0	<90.0	Poor
1.0–2.0	>90.0–99.0	Satisfactory
2.0–3.0	>99.0–99.9	Good
>3.0	>99.9	very good

## Data Availability

All data is contained within the manuscript.
